# Prospective assessment of the quality of life before, during and after image guided intensity modulated radiotherapy for prostate cancer

**DOI:** 10.1186/s13014-016-0689-4

**Published:** 2016-09-07

**Authors:** Joen Sveistrup, Ole Steen Mortensen, Jakob B Bjørner, Svend Aage Engelholm, Per Munck af Rosenschöld, Peter Meidahl Petersen

**Affiliations:** 1 Department of Oncology, Rigshospitalet, Copenhagen, Denmark; 2 Department of Occupational Medicine, Holbæk Hospital, Holbæk, Denmark; 3 National Research Centre for the Working Environment, Copenhagen, Denmark

**Keywords:** Prostate cancer, Radiotherapy, Quality of life, Questionnaire

## Abstract

**Background:**

Radiotherapy (RT) in combination with androgen deprivation therapy (ADT) for prostate cancer (PCa) carries a risk of gastrointestinal (GI) and genitourinary toxicity, which might affect the quality of life (QoL). The purpose of this study was to assess the QoL in patients with PCa before, during and after radiotherapy (RT) and to compare the QoL 1 year after RT to a normal population.

**Methods:**

The QoL was evaluated prospectively by the self-administered questionnaire SF-36 in 87 patients with PCa. The SF-36 was completed before RT (baseline), at start of RT, at end of RT and 1 year after RT. A mixed model analysis was used to determine the changes in QoL at each time point compared to baseline. The patients’ QoL 1 year after RT was compared to a normal population consisting of 462 reference subjects matched on age and education.

**Results:**

One year after RT, patients reported significantly less pain and significantly fewer limitations due to their physical health compared to baseline. Compared to the normal population, patients reported significantly less pain 1 year after RT. However, patients also reported significantly less vitality, worse mental health as well as significantly more limitations due to physical and mental health 1 year after RT compared to the normal population.

**Conclusions:**

In this study, patients with PCa did not experience significant impairment in the QoL 1 year after RT compared to baseline. However, patients reported significantly worse mental health before, during and 1 year after RT compared to the normal population.

## Background

Radiotherapy (RT) in combination with androgen deprivation therapy (ADT) is an established treatment for prostate cancer (PCa). Two large randomised trials have shown that RT in combination with ADT results in significantly improved overall survival and PCa-specific survival in patients with locally-advanced PCa compared to ADT alone [1, 2].

The introduction of image guided intensity modulated radiotherapy (IG-IMRT) has resulted in decreased toxicity due to higher accuracy of the dose delivery [3]. However, prospective longitudinal investigations of the QoL following new radiation techniques such as IG-IMRT are few. Retrospective analyses of QoL are biased by the time delay and often baseline values are not taken into account [4].

We have performed a prospective analysis of the urinary, gastrointestinal (GI) and sexual symptoms as well as the QoL before, during and after IG-IMRT in a cohort of 87 patients with PCa. The toxicity results have been published previously [5]. Here, we report the changes in QoL at the start of RT, at the end of RT and 1 year after RT compared to baseline. Furthermore, we compare the patients’ QoL 1 year after RT to a normal population matched on age and education.

## Methods and materials

### Patients

A total of 87 consecutive patients with PCa referred to curative RT at the Department of Oncology at Rigshospitalet in Copenhagen, Denmark were included in the study. Information about patient and disease characteristics was obtained from the patient records. The cohort has been described previously [5]. Patient and disease characteristics are found in Table 1. The median age was 67 (range 50–75). The median prostate specific antigene (PSA) level was 11 ng/ml (range 3.1–91), and 75 % of the patients had a T-stage of T2c or higher. Seventy-three patients (84 %) had high risk disease according to the d’Amico classication [6], and the remaining 14 patients (16 %) had intermediate risk disease. Twenty-one patients (24 %) had one or more comorbidities with obesity, hypertension and chronic obstructive pulmonary disease as the most frequent. There were no signs of metastatic disease in any of the patients based on a CT of the abdomen and a bone scintigraphy.

**Table 1:** Patient and disease charateristics

Categorical characteristic	No. patients *n* = 87	Percent
T-stage		
≤ T2a	17	19
T2b	5	6
≥ T2c	65	75
Gleason Score		
6	12	14
7	49	56
≥ 8	26	30
Risk group		
Low	0	0
Intermediate	14	16
High	73	84
Type of ADT		
GnRH agonist	86	99
Antiandrogen	1	1
Continuous characteristic	Median	Range
PSA (ng/ml)	11	3.1–91.0
Age (yr)	67	50–75

All patients received neo-adjuvant and concomitant androgen deprivation therapy (ADT). The 73 patients with high risk disease received ADT for 3 years while the 14 patients with intermediate risk disease received ADT for 6 months.

### Radiotherapy

The radiotherapy has been described in details previously [7]. In brief, the RT was delivered with a single beam rotational technique (VMAT - RapidArc®, Varian Medical Systems, Palo Alto, US). For RT planning, three fiducial markers were implanted in the prostate through the rectum under ultrasound guidance. The CTV was defined as the prostate and, if present, extracapsular tumor. The proximal 2 cm of the seminal vesicles (SM) were included in the CTV if the risk of SM involvement exceeded 10 % [8]. The PTV margin was 5 mm in the right-left and anterior-posterior plane and 7 mm in the superior-inferior plane.

Image registrations and plans were based on ICRU guidelines and performed with the Eclipse treatment planning system (Varian Medical Systems, Palo Alto, CA, US).

The dose was 78 Gy (2 Gy pr. fraction, 5 fractions pr. week).

### Questionnaire

We used a combination of two existing questionnaires. The quality of life was measured with the validated Danish version of SF-36 [9]. The toxicity was measured with the validated PCa-specific Prostate Cancer Symptom Scale (PCSS) questionnaire [10].

SF-36 consists of 36 questions about physical and mental health. Based on the responses eight scales are created. Four of the scales measure the physical function: Physical Functioning, Role Physical - role limitations due to physical problems, Bodily pain and General Health. Another four scales measure the mental function: Vitality, Social Functioning, Role Emotional – role limitations due to emotional problems and Mental Health. Each scale is scored from 0 to 100 with higher scores indicating better function. A change in score of 10 points or more is generally considered clinically significant [11]. The physical and mental scales are furthermore transformed into two summarized measures: the Physical Component Scale (PCS) and the Mental Component Scale (MCS).

The PCSS contains questions about specific urinary, GI and sexual symptoms. The questions are answered on a linear scale from 0 to 10, where 0 indicates “no problems” and 10 indicates a “high degree of problems”. In the present analysis we used the following three questions from the PCSS, “Do you have any urinary/GI/sexual bother?” in order to determine the impact of urinary, GI and sexual bother on the SF-36 scores.

The questionnaires were completed before RT (baseline), at the start of RT, at the end of RT and 1 year after RT. Of the 87 patients, 84 (97 %) completed the questionnaire at baseline, 80 (92 %) completed the questionnaire at the start of RT, 77 (89 %) completed the questionnaire at the end of RT and 77 (89 %) completed the questionnaire 1 year after RT.

For each of the patients who completed the final questionnaire, six reference subjects matched on age and education were randomly chosen from a normal population data set, which contains SF-36 scores from approximately 3000 Danish individuals.

### Statistics

A mixed model analysis of repeated measurements was used to determine the mean changes in SF-36 scores at the start of RT, at the end of RT and 1 year after RT compared to baseline. The following covariates were applied in the analysis: age, smoking, comorbidity, the use of ADT, urinary bother, GI bother and sexual bother.

The Mann-Whitney test was used to compare the patients’ SF-36 scores 1 year after RT to the normal population’s SF-36 scores. For the normally distributed scales PCS and MCS an independent-samples *T* test was used. All analyses were performed with SPSS v. 19.0.

## Results

### QoL scores

Table 2 lists the mean changes in SF-36 scores at the start of RT, at the end of RT and 1 year after RT compared to baseline. Furthermore, the impact of the covariates is listed. For the continuous variables *age*, *urinary bother*, *GI bother* and *sexual bother* the results indicate the mean change in SF-36 score per one unit increase in the variables.

**Table 2:** Estimated changes in SF-36 scores from the start of RT, the end of RT and 1 year after RT compared to baseline are listed on the left. The impact of the covariates on the scores is listed on the right

Scale	Time			Covariates						
	Mean change from baseline (95 % CI)			Mean change (95 % CI)						
	Start RT	End RT	1 year after RT	Age	Comorb.	Smok.	ADT	Urinary bother	GI bother	Sexual bother
Physical functioning	−0.7 (−3.6;2.2)	−3.8 (−6.7; −0.9)	−3.6 (−6.5; −0.7)	−0.2 (−0.7;0.3)	−7.0 (−13.8; −0.3)	−0.5 (−7.8;6.8)	−2.1 (−7.7;3.5)	−0.6 (−1.1; −0.1)	0.1 (−0.6;0.7)	0.2 (−0.2;0.6)
	*p* = 0.638	*p* = 0.010*	*p* = 0.015*	*p* = 0.387	*p* = 0.041*	*p* = 0.897	*p* = 0.464	*p* = 0.014*	*p* = 0.865	*p* = 0.357
Role physical	13.3 (4.0;22.6)	1.5 (−7.9;11.0)	11.4 (2.0;20.8)	−0.2 (−1.4;1.0)	−7.2 (−22.8;8.4)	−1.2 (−18.1;15.8)	−6.8 (−24.5;10.9)	−3.3 (−4.8; −1.9)	−1.0 (−2.9;1.0)	0.1 (−1.1;1.3)
	*p* = 0.005*	*p* = 0.754	*p* = 0.018*	*p* = 0.741	*p* = 0.362	*p* = 0.890	*p* = 0.450	*p* < 0.001*	*p* = 0.320	*p* = 0.873
Bodily pain	11.9 (6.1;17.7)	0.0 (−5.9;5.8)	11.4 (5.5;17.3)	−0.2 (−0.8;0.4)	0.8 (−7.4;9.1)	−2.6 (−11.6;6.3)	2.1 (−8.7;12.9)	−1.4 (−2.3; −0.5)	−2.0 (−3.2; −0.9)	0.0 (−0.7;0.7)
	*p* < 0.001*	*p* = 0.990	*p* < 0.001*	*p* = 0.558	*p* = 0.840	*p* = 0.562	*p* = 0.698	*p* = 0.002*	*p* = 0.001*	*p* = 0.987
General health	0.6 (−2.5;3.8)	−2.0 (−5.2;1.2)	−3.6 (−6.8; −0.3)	0.5 (0.0;1.1)	−8.4 (−15.8; −1.0)	0.4 (−7.6;8.3)	−5.7 (−11.9;0.6)	−0.6 (−1.1;0.0)	−1.0 (−1.7; −0.3)	0.1 (−0.3;0.5)
	*p* = 0.687	*p* = 0.222	*p* = 0.031*	*p* = 0.071	*p* = 0.026*	*p* = 0.931	*p* = 0.075	*p* = 0.040*	*p* = 0.004*	*p* = 0.588
PCS	2.1 (0.5;3.7)	−1.1 (−2.7;0.5)	0.7 (−0.9;2.3)	−0.1 (−0.3;0.2)	−2.0 (−5.3;1.3)	0.5 (−3.0;4.1)	−1.7 (−4.7;1.3)	−0.5 (−0.8; −0.3)	−0.4 (−0.7;0.0)	0.2 (0.0;0.4)
	*p* = 0.008*	*p* = 0.181	*p* = 0.392	*p* = 0.584	*p* = 0.225	*p* = 0.763	*p* = 0.270	*p* < 0.001*	*p* = 0.032*	*p* = 0.113
Vitality	2.9 (−1.0;6.9)	−4.4 (−8.4; −0.4)	1.3 (−2.7;5.3)	0.6 (−0.1;1.3)	−7.3 (−16.5;1.8)	−5.5 (−15.4;4.4)	0.1 (−7.6;7.8)	−1.1 (−1.7; −0.4)	−1.0 (−1.8; −0.1)	−0.2 (−0.8;0.3)
	*p* = 0.144	*p* = 0.030*	*p* = 0.517	*p* = 0.087	*p* = 0.113	*p* = 0.271	*p* = 0.979	*p* = 0.001*	*p* = 0.028*	*p* = 0.378
Social functioning	3.0 (−0.9;6.9)	−2.3 (−6.3;1.7)	3.7 (−0.3;7.7)	0.4 (−0.2;0.9)	−4.8 (−11.6;2.1)	2.6 (−4.9;10.0)	4.9 (−2.8;12.6)	−0.5 (−1.1;0.1)	−1.3 (−2.1; −0.5)	−0.3 (−0.8;0.2)
	*p* = 0.134	*p* = 0.261	*p* = 0.068	*p* = 0.171	*p* = 0.170	*p* = 0.495	*p* = 0.210	*p* = 0.132	*p* = 0.003*	*p* = 0.240
Role emotional	4.6 (−3.2;12.5)	−0.1 (−8.1;7.8)	2.1 (−5.8;10.1)	0.3 (−0.8;1.3)	−6.6 (−20.4;7.3)	−4.4 (−19.4;10.6)	−2.2 (−17.3;12.9)	−1.4 (−2.7; −0.2)	−0.9 (−2.6; −0.2)	−0.8 (−1.8;0.2)
	*p* = 0.248	*p* = 0.972	*p* = 0.598	*p* = 0.597	*p* = 0.348	*p* = 0.565	*p* = 0.773	*p* = 0.028*	*p* = 0.266	*p* = 0.099
Mental health	5.1 (1.5;8.8)	2.0 (−1.7;5.7)	6.3 (2.6;10.0)	0.3 (−0.2;0.8)	−5.7 (−12.7;1.3)	−7.1 (−14.7;0.4)	2.8 (−4.4;9.9)	−0.8 (−1.4; −0.2)	−0.6 (−1.4;0.2)	−0.3 (−0.8;0.1)
	*p* = 0.006*	*p* = 0.282	*p* = 0.001*	*p* = 0.242	*p* = 0.107	*p* = 0.065	*p* = 0.445	*p* = 0.014*	*p* = 0.129	*p* = 0.148
MCS	1.7 (−0.3;3.8)	0.1 (−2.0;2.1)	2.1 (0.0;4.1)	0.3 (0.0;0.5)	−2.8 (−6.6;1.0)	−2.6 (−6.7;1.5)	1.4 (−2.5;5.4)	−0.3 (−0.7;0.0)	−0.4 (−0.9;0.0)	−0.3 (−0.6;0.0)
	*p* = 0.091	*p* = 0.931	*p* = 0.047*	*p* = 0.072	*p* = 0.149	*p* = 0.208	*p* = 0.473	*p* = 0.052	*p* = 0.055	*p* = 0.026*

*RT* Radiotherapy

*PCS* Physical Component Scale

*MCS* Mental Component Scale

*Statistically significant

The only clinically significant changes (change >10 points) were observed in the physical domain. Patients experienced significantly less pain and significantly fewer limitations due to physical health both at the start of RT and at the end of RT compared to baseline. However, there was a significant decrease in the physical functioning at the end of RT and 1 year after RT with mean changes of −3.8 (−6.7; −0.9) and −3.6 (−6.5; −0.7), respectively. The PCS increased significantly at the start of the RT compared to baseline but no significant changes were observed at the end of RT or 1 year after RT.

In the mental domain, the mental health increased significantly at the start of RT and 1 year after RT with mean changes of 5.1 (1.5;8.8) and 6.3 (2.6;10.0), respectively. Accordingly, the MCS increased significantly 1 year after RT compared to baseline.

Comorbidity was associated with a significant deterioration in the physical functioning and general health scores with mean changes of −7.0 (−0.3; −13.8) and −8.4 (−1.0; −15.8), respectively.

Increasing urinary bother was associated with decreased QoL in eight of the 10 scales, whereas increasing GI bother was associated with decreased QoL in five scales. Finally, increasing sexual bother was associated with decreased QoL in one scale.

### Comparison with the normal population

**Fig. 1:**
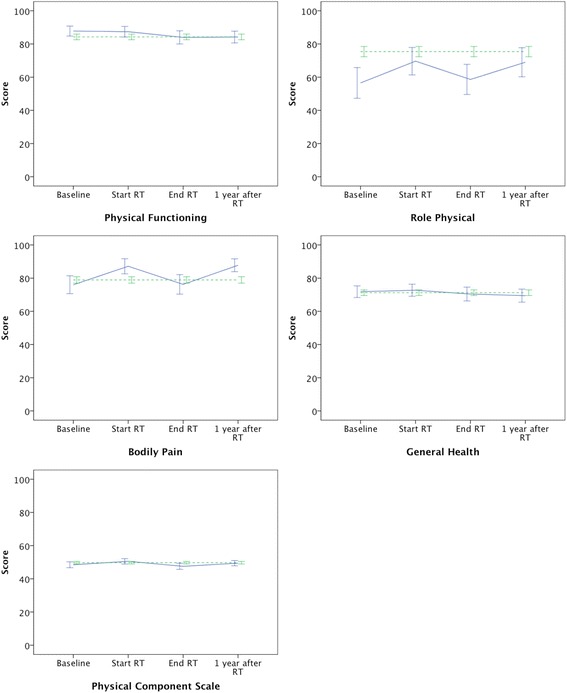
The graphs show the five scales in the physical domain with mean scores and 95 % confidence intervals for patients (*solid line*) at baseline, at the start of RT, at the end of RT and 1 year after RT as well as the mean scores for the normal population (*dashed line*)

**Fig. 2:**
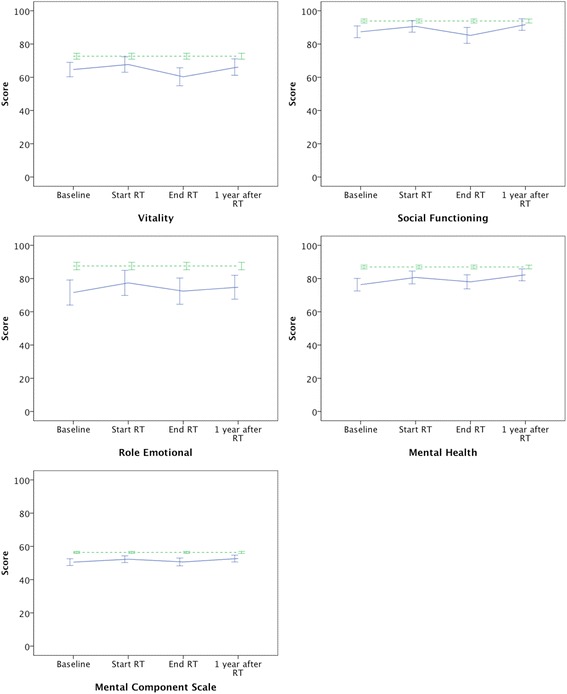
The graphs show the five scales in the mental domain with mean scores and 95 % confidence intervals for patients (*solid line*) at baseline, at the start of RT, at the end of RT and 1 year after RT as well as the mean scores for the normal population (*dashed line*)

The mean SF-36 scores with 95 % confidence intervals for patients and the normal population are shown in Figs. 1 and 2. The SF-36 scores for the normal population are displayed throughout the entire study period for comparison although the values represent one measurement only corresponding to 1 year after RT.

Table 3 compares the SF-36 scores between the 77 patients who completed the questionnaire 1 year after RT and the normal population consisting of 462 reference subjects. The normal population reported a significantly higher mental QoL in terms of more vitality, fewer limitations due to mental health, better mental health and finally a higher MCS score. Furthermore, the normal population reported significantly fewer limitations due to physical problems. Oppositely, patients reported significantly less pain 1 year after RT compared to the normal population.

**Table 3:** SF-36 scores for the patient group 1 year after RT compared to the normal population

SF scale		Mean (95 % CI)	*p* value
Physical functioning	Patient	83.62 (80.01–87.23)	0.247
	Normal	84.25 (82.44–86.07)	
Role physical	Patient	67.43 (58.44–76.43)	0.024*
	Normal	76.25 (72.91–79.58)	
Bodily pain	Patient	87.30 (83.33–91.68)	0.002*
	Normal	78.65 (76.55–80.75)	
General health	Patient	67.80 (63.51–72.09)	0.058
	Normal	71.35 (69.52–73.19)	
PCS	Patient	49.24 (47.63–50.84)	0.583
	Normal	49.74 (48.90–50.58)	
Vitality	Patient	64.74 (59.86–69.91)	0.001*
	Normal	72.31 (70.40–74.24)	
Social functioning	Patient	91.12 (87.57–94.67)	0.039
	Normal	94.13 (92.86–95.40)	
Role emotional	Patient	73.68 (66.40–80.97)	<0.001*
	Normal	88.02 (85.67–90.38)	
Mental health	Patient	81.25 (77.64–85.86)	0.001*
	Normal	87.08 (85.87–88.28)	
MCS	Patient	52.18 (50.10–54.26)	<0.001*
	Normal	56.46 (55.83–57.09)	

*PCS* Physical Component Scale

*MCS* Mental Component Scale

*Statistically significant

## Discussion

In this study, we investigated the changes in QoL in patients with PCa following IG-IMRT. The QoL was evaluated prospectively with the use of a self-administered questionnaire and the impact of various factors on the QoL was determined. Furthermore, we compared the QoL 1 year after RT to the QoL of a normal population matched on age and education.

The results in this study display a rather complex pattern of changes in the QoL during and after RT. Overall, we did not observe any clinically significant impairment in the QoL 1 year after RT compared to baseline. This is in line with previously published results [12, 13]. However, it is worth noticing that the physical functioning and general health decreased significantly 1 year after RT, even though the decreases were small (<5 points) and not considered clinically significant. In contrast, patients reported significantly fewer limitations due to physical problems and significantly less pain at the start of RT and 1 year after RT compared to baseline (change >10 points). The increase in QoL at the start of RT might be explained by a concept called benefit finding. Benefit finding refers to the fact that stressful events such as serious illness can result in a new more positive attitude and appreciation of ones own strength [14].

The patients’ QoL 1 year after RT was significantly different from the normal population in six of the 10 scales. In the physical domain, patients reported significantly less pain 1 year after RT compared to the normal population. Mols et al. [15] compared PCa survivors with a normal population and reported similar result for patients treated with radical prostatectomy, RT and watchful waiting. The exact reason for the lower degree of pain in patients with PCa following treatment is uncertain. We do not know the level of pain in the patients before they were diagnosed with PCa. It may have been comparable to the level observed 1 year after RT and as such lower than the level of pain in the normal population. Patients with PCa who are treated with RT are positively selected, since they must be without disabling comorbidity and have a remaining life expectancy of more than 10 years. As a consequence, they might have less comorbidity than the normal population, and this might translate into less pain.

Conversely, patients reported significantly worse mental health 1 year after RT compared to the normal population. The graphs in Fig. 2 illustrate that the SF-36 scores in the mental domain are lower in patients than in the normal population at all times, including baseline. Again, we have no information about the patients’ mental QoL before they were diagnosed with PCa, but it is likely that the mental QoL has deteriorated as a consequence of the psychological distress associated with the diagnosis of cancer [16]. Furthermore, most patients had commenced ADT before they completed the baseline questionnaire. Therefore, the lower mental QoL in patients at baseline might be caused by the diagnosis of cancer and the use of ADT, or a combination of both. Sanda et al. [17] demonstrated that vitality was lower in patients who received ADT and that this effect persisted for up to 2 years despite the fact that the majority of patients received ADT for less than 1 year. This might also be the reason why the use of ADT did not have an impact on any of the SF-36 scores in our study. Furthermore, there was no significant difference in the QoL 1 year after RT between high risk patients (3 years of ADT) and intermediate risk patients who no longer received ADT 1 year after RT (results not shown).

We also determined the impact of urinary, GI and sexual bother on the QoL. Increasing urinary and GI bother were associated with decreased QoL in several scales. Clark et al. [18] also evaluated the impact of symptoms on SF-36 scores and found similar results. As a consequence, further reduction of the urinary and GI toxicity following RT for PCa might prove beneficial to the patients’ QoL. Oppositely, increasing sexual bother was only associated with a worse MCS score indicating an overall small impact of sexual bother on the QoL.

Comorbidity was associated with a significant deterioration in two of the five physical scales. The fact that comorbidity only seemed to affect the patients’ physical functioning and general health probably reflects the rather low level of comorbidity in patients with PCa who are treated with RT.

This study has some limitations. The patients were only followed for 1 year after RT. The results might change after longer follow-up, especially since the toxicity might develop further even after 1 year after RT. Furthermore, the number of patients in this study was relatively low, which to some degree affects the reliability of the findings.

## Conclusion

This study demonstrates that IG-IMRT does not lead to clinically significant deteriorations in the QoL 1 year after RT. Compared to a normal population, patients reported significantly less pain which might reflect the positive selection of patients with PCa treated with radiation. However, we also found that patients reported significantly worse mental health before, during and 1 year after RT compared to the normal population, which in addition to the radiation might be a result of both the diagnosis of PCa as well as the use of ADT.

## Abbreviations

ADT: Androgen deprivation therapy
GI: Gastrointestinal
IG-IMRT: Image guided intensity modulated radiotherapy
MSC: Mental component scale
PCa: Prostate cancer
PCS: Physical component scale
PSA: Prostate specific antigene
QoL: Qulaity of life
RT: Radiotherapy
VMAT: Volumetric modulated arc therapy
